# Interfered feature elimination coupled with feature group selection for wound infection detection by electronic nose

**DOI:** 10.1371/journal.pone.0327748

**Published:** 2025-07-10

**Authors:** Jia Liu, Jinglei Zhang, Shaoqi Zhang, Kaiwei Li, Xiang Li, Shuo Zhang, Hang Gu, Zhen Chen, Chao Liu, Nan Zhang, Tong Sun

**Affiliations:** 1 School of Computer Science and Technology, Zhengzhou University of Light Industry, Zhengzhou, China; 2 The First Affiliated Hospital of Sun Yat-sen University of Clinical and basic research of assisted reproductive technology, Guangzhou, China; 3 College of information and management science, Henan Agricultural University, Zhengzhou, China; 4 The First Affiliated Hospital of Henan University of CM Clinical and Basic Research on the Treatment of Digestive Tract Tumors with Integrative Medicine, Zhengzhou, China; University of Kelaniya, SRI LANKA

## Abstract

As the precise odor-sensing equipment, the electronic nose integrates multiple advanced and sensitive sensors that can identify wound infections non-invasively and rapidly by analyzing wound characteristic odor. To reduce the cost of sensors and improve or maintain e-nose’s performance, efficient optimization of sensor arrays is required. For this issue, we proposed a new sensor array optimization algorithm named Interfered Feature Elimination coupled with Feature Group Selection (IFE-FGS). In this method, the IFE algorithm first removed the bad sensor features; then the FGS algorithm determined the optimized sensor combination by gradually selecting the features in groups. The experimental results show the superiority of the IFE- FGS method on two bacteria datasets and six public gene expression profiling datasets. IFE- FGS achieves the classification accuracy of 93.95% and 94.94% in mean accuracy and max accuracy, respectively, on the bacteria dataset 2, which is significantly ahead of the comparison methods. Besides, our proposed method shows consistency and effectiveness. It achieves excellent performance, which takes two first-places, four second-places in mean accuracy, one first-place, and six second-places in max accuracy. Moreover, it also explores three novel and valuable discoveries for the electronic nose: 1) It can effectively identify biomarkers in the application. 2) It can effectively distinguish the degree of chemical components contributing to odors. 3) It can reveal the effective detection range of the targets.

## 1. Introduction

Wound bacterial infection is a common complication of wounds. It can severely hinder wound healing, make the wound difficult to treat, and increase the risk of amputation and death to the patients [1–3]. The traditional detection methods, such as morphological examination and immunological test, have the disadvantages of high expenses, long detection time, invasive injury, etc., resulting in the standard cycle of diagnosis and treatment may last for several months, which cannot meet the actual needs of better diagnosis and treatment [4,5]. In-home care, the patient and caregiver have difficulty being aware of wound changes in time, which can easily cause the condition to be delayed. Therefore, wound bacterial infections have seriously threatened human health and brought a substantial economic burden on society [6–8]. Based on the above needs, how to accurately, quickly, and low-cost detection of wound bacterial infection has attracted the attention of many researchers.

The Odor-sensing approach was able to identify wound infections by analyzing wound characteristic odor, which has the advantages of noninvasiveness, rapid response, easy operation, and etc., and it is very suitable for routine detection of wound infection [9,10]. Electronic nose (e-nose) is a common odor-sensing approach, which is an electronic device that mimics the working principle of the mammalian olfactory system [11–15] and has been intensively applied in many fields such as medical diagnosis [16–19], food safety [20], flavor identification [21–23], environmental monitoring [24–26]. An e-nose system typically consists of three parts: a multi-sensor array to sense the target odor, a signal processing unit to generate odor data, and a set of dedicated algorithms to identify the odor. Bacterial wound infection detection uses gas sensors with broad selectivity and cross-sensitivity to jointly construct odor fingerprints. Finally, it recognizes the sensor signal through the dedicated odor recognition algorithm. Hence, E-nose can quickly and effectively detect and monitor wound infection by analyzing wound characteristic odor.

For the established electronic nose sensor array, the performance of the algorithm applied to odor recognition determines the final detection capability of the whole electronic nose system. However, the known odorous compounds are as many as more than 10,000 [27], so developing a universal e-nose to complete all the odor detection missions is nothing but wishful thinking. Therefore, to achieve rapid and effective detection of bacterial wound infection, it is necessary to design a specific sensor array optimization algorithm to reduce the cost and even improve the performance of the electronic nose system.

Based on the above research requirements, sensor array optimization (SAO) algorithms are increasingly gaining attention in the electronic nose community. The general concept of SAO is to determine the best sensor combination to provide effective and robust odor judgment. This implies that the SAO algorithm should guide us in eliminating poor and redundant sensors and/or adding necessary sensors in the array, thus achieving rapid detection of bacterial wound infection while reducing the cost of sensors. Currently, in the e-nose community, SAO is commonly solved by introducing feature selection technology in the machine learning community. The technology introduction could be crystallized into two major technical issues, i.e., 1) the evaluation of the features and 2) the searching scheme to form the compact key feature set. For 1), statistic and information theory methods are usually adopted, such as Pearson correlation coefficient, mutual information, and performance sensitivity. For 2), Sequential Selection Algorithms and Heuristic Search Algorithms have been extensively applied, such as Sequential Feature Selection (SFS), Sequential Floating Forward Selection (SFFS), Particle Swarm Optimization (PSO), and Genetic Algorithm (GA). The details about this can refer to the review article by Chandrashekar and Sahin [28] and other literature [29–31].

Some advanced methods applied for medical research have demonstrated the superiority of their algorithms and are capable of effectively extracting valuable information. To be specific, Elnaz Pashaei [32] proposed a novel Binary Sand Cat Swarm Optimization to solve the “curse of dimensionality” in biomedical data analysis and can even successfully classify colon cancer without any error [33]. combined hub gene ranking techniques and feature selection algorithms to identify reliable biomarkers and therapeutic targets for Alzheimer’s Disease research [34]. Integrated differential expression with network centrality analysis and then identified genes over-represented in crucial pathways and cancer fitness genes [35]. used metaheuristic algorithm can improve classification in several publicly available high-dimensional biomedical datasets. Although these methods did not focus on the research field of the electronic nose community, they still brought us many valuable inspirations.

In some released works of SAO, for example, in [36–43], individual evaluation and manipulation of a single feature form the basis of the optimization process. However, we believe that this ignored the working principle of e-nose, i.e., using a set of sensors with broad selectivity and cross-sensitivity to identify odors, which may lead to performance loss. Therefore, we infer that the e-nose sensors can be viewed as working in several groups. Inspired by this, we propose a novel SAO searching scheme named Feature Group Selection (FGS), which is used in our application of bacteria detection. In our practice, original features are first filtered by the Interfered Feature Elimination (IFE) algorithm in an unsupervised way to exclude the features with a low signal-noise ratio (SNR), and then the FGS algorithm is employed to find the key and compact feature group.

This paper aims to conduct research on SAO for e-nose and optimize the custom e-nose for detecting wound infection. The main work is threefold: 1) A feature evaluation criterion based on linear transformation, which was widely used in feature selection, is reformulated; 2) Under a reasonable assumption, the IFE method is presented to exclude the features with low SNR; 3) We proposed an effective and efficient sensor arrays optimization method to optimize the custom e-nose for wound infection detection and analysis.

For the arrangement of this paper, before elaborating on the FGS algorithm, the feature evaluation criterion and IFE algorithm are introduced, which are used to preprocess the original features. Specifically, the textual construction and content are as follows. Section 2.1 presents a brief description of the custom e-nose, the measurement process of 3 common pathogenic bacteria, and two custom bacteria datasets. In section 2.2, a feature evaluation criterion based on linear transformation is first reviewed and then reformulated, which is used in the IFE algorithm. In section 2.3, the IFE method is presented based on a reasonable assumption and employment of the criterion and Pearson correlation coefficient. Subsequently, in section 2.4, the FGS algorithm is elaborated step by step, where a crossover selection and a mutation selection are involved. In section 2.5, the validation protocol of the involved algorithms is presented. After describing the initial performance of the e-nose without SAO in section 3.1, the validation results of the proposed method and eight benchmarks on the bacteria datasets and six public datasets are discussed in section 3.2, where the reason for the outstanding merit of the IFE-FGS method on model stability is explained. In section 3.3, we seek some knowledge extension on the application with the assistance of the SAO algorithm. Finally, the conclusion of the whole paper is made in section 4.

## 2. Materials and methods

### 2.1. Experiment and datasets

In this research, we further investigated the potential of an odor-sensing approach for the detection of wound infections by proposing a novel sensor array optimization algorithm. Specifically, we collect the data from the experiments of 44 Sprague Dawley (SD) rats [42,44]. Besides, based on a full-thickness wound infection model, the rats were inoculated with three common wound infection pathogens, including Escherichia-coli (EC), Pseudomonas aeruginosa (PA), and Staphylococcus aureus (SA), for three infected groups and sterile Phosphate Buffer Solution (PBS) for a control group, respectively. The prototype was used to directly sniff the rat wound samples without any sample pretreatment to acquire the wound odor data, and an information fusion algorithm dedicated to the dual odor-sensing prototype was developed to accomplish the final identification of the rat infection type.

The specifications of the sensors used in the e-nose and a description of the two bacteria datasets were listed in Tables 1 and 2, respectively.

**Table 1:** Specifications of the involved sensors of the custom e-nose.

No.	Sensor	Target/Sensitive chemicals	Type	Producer (Country)
1	SHT-10	Temperature	digit	Sensirion (Switzerland)
2	SHT-10	Humidity	digit	Sensirion (Switzerland)
3	MS5611	Gas pressure	digit	Measurement (Switzerland)
4	CHV-25P	voltage	digit	Sse (China)
5	NH3-3E100SE	Ammonia	Electrochemistry	CITY (UK)
6	MS1100	Toluene, benzene, formaldehyde, VOCs	MOS	Ogam (Korea)
7	TGS2611C	Methane, ethane, carbon monoxide and other combustible gases	MOS	Figaro (Japan)
8	MQ137	Ammonia, hydrogen, ethanol	MOS	Winsen (China)
9	TGS4161	Carbon dioxide	Electrochemistry	Figaro (Japan)
10	TGS2600	Air pollutants, cigarette smoke, methane, ethanol	MOS	Figaro (Japan)
11	MP4	Combustible gas, methane, methane, etc.	MOS	Winsen (China)
12	MP135A	Ethanol, cigarette smoke, air pollutants	MOS	Winsen (China)
13	TGS2610C	Methane, ethane and other combustible gases	MOS	Figaro (Japan)
14	MQ135	Ammonia, sulfide, benzene	MOS	Winsen (China)
15	MQ3B	Ethanol, with antihydrogen and carbon monoxide interference	MOS	Winsen (China)
16	TGS2610D	Same as TGS2610C (increase alcohol filtration), methane, ethane, propane, butane	MOS	Figaro (Japan)
17	4OXV	Oxygen	Electrochemistry	CITY (UK)
18	TGS822	Organic solvents such as methanol, ethanol, etc.	MOS	Figaro (Japan)
19	4S	Sulfur dioxide	Electrochemistry	CITY (UK)
20	GSBT-11	VOCs, toluene, benzene, formaldehyde	MOS	Ogam (Korea)
21	MQ136	Hydrogen sulfide, carbon monoxide, methane	MOS	Winsen (China)
22	SP3-AQ2–01	VOCs, methane, carbon monoxide, hydrogen	MOS	FIS (Japan)
23	TGS813	Methane, propane, ethanol, hydrogen, carbon monoxide	MOS	Figaro (Japan)
24	CH2O/M-10	Formaldehyde	Electrochemistry	Membrapor (Switzerland)
25	TGS816	Methane, propane, methane, carbon monoxide, hydrogen, ethanol, combustible gas	MOS	Figaro (Japan)
26	MQ138	Alcohols, ketones, aldehydes, aromatic and other organic solvents	MOS	Winsen (China)
27	TGS2620	VOCs, ethanol, organic solvent vapor	MOS	Figaro (Japan)
28	MP503	Alcohol, smoke, isobutyl alcohol, formaldehyde	MOS	Winsen (China)
29	4HS	Hydrogen sulfide	Electrochemistry	CITY (UK)
30	TGS826	Ethanol, isobutene, high sensitivity to ammonia	MOS	Figaro (Japan)
31	MP901	Alcohol, smoke, formaldehyde, toluene, benzene, acetone	MOS	Winsen (China)
32	WSP2110	Ethanol, benzene, toluene, hydrogen, formaldehyde, VOCs	MOS	Winsen (China)
33	TGS2611D	Same as TGS2611C, increase alcohol filtration	MOS	Figaro (Japan)
34	TGS2602	Air pollutants, VOCs, ammonia, hydrogen sulfide	MOS	Figaro (Japan)

**Table 2:** Description of the two custom bacteria odor datasets.

	Sample 1 (EC)	Sample 2 (SA)	Sample 3 (PA)	Sample 4 (LB)	Sample 5 (SA, PA)	Sample 6 (EC, SA)	Sample 7 (EC, PA)	Sample 8 (EC, SA, PA)
**Dataset 1** (carrier gas of clean air, 428 samples)	55	56	46	72	44	49	47	59
**Dataset 2** (carrier gas of volatiles of medical ethanol, 320 samples)	32	34	33	48	39	34	32	68

The custom e-nose consisted of two modules: the gas channel module and the signal processing and control module. The gas channel module mainly included Teflon gas lines, an air filter (Teflon membrane), a single-way solenoid valve and two three-way solenoids, a Teflon sample chamber, a sensor array of 4 environmental sensors and 30 gas sensors, a mass flow controller (MFC), and a vacuum pump. The signal processing and control module mainly included an upper computer, a signal processing circuit, and a control board. The detection process has 14 min, including baseline collecting of 3 min, sample collecting of 3 min, system purging of 8 min, where the gas flow rate is set at 100 ml/min by the MFC.

Escherichia coli (EC), Staphylococcus aureus (SA), and Pseudomonas aeruginosa (PA) are three common pathogenic bacteria responsible for wound infection. By using different carrier gas, we detected the three bacterial fluids by the custom e-nose, and acquired two bacteria datasets. Dataset 1 was collected using clean air as the carrier gas, and dataset 2 using the volatiles of medical ethanol as the carrier gas to simulate the odor background of the hospital ward.

### 2.2. Evaluation of feature relevance by linear transformation

The feature evaluation criterion serves as the assessment of feature relevance for the SAO algorithm. There exists a kind of feature evaluation criteria derived from linear transformation, such as Principal Component Analysis (PCA), Linear Discriminant Analysis (LDA), and Independent Component Analysis (ICA), which has been widely used to estimate feature relevance [42,45–51]. In this subsection, the fundamental criteria was first introduced, and a brief review of them was listed in Table 3. And then they were corrected and reformulated.

**Table 3:** Previously literatures on feature evaluation for relevance based on linear transformation.

Ref.	Year	Mapping	Authors	Evaluation criterion	Description
[42]	2017	PCA/LDA	Sun Hao et al.	$I_{j}=\left|\sum {\mathrm{\backslash nolimits}}_{i=1}^{d}e_{i,j}\right|$ , (*d* = *s*)	Five feature selection methods were investigated on bacteria culture dataset, wherein Wilks’ Λ-statistic and LDA-based method showed good effect respectively, but not the PCA-based method.
[45]	2004	PCA	Arnaz Malhi et al.	$I_{j}=\left|e_{1,j}\right|$	A PCA-based feature selection for machine defect classification was proposed where the feature importance was considered only on the first projection direction.
[47]	2008	PCA	Jun-Ling Xu et al.	$I_{j}=\sum {\mathrm{\backslash nolimits}}_{i=1}^{d}\lambda_{i}\left|e_{i,j}\right|$, (*d* ≤ *s*)	PCA was used to rank features and then, for clustering, key features set was determined by FS. The affection of dimension *d* of mapped space was discussed. Effectiveness was confirmed on ten open datasets.
[50]	2014	Regularized LDA	Alok Sharma et al.	$I_{j}=\sum {\mathrm{\backslash nolimits}}_{i=1}^{d}\sum {\mathrm{\backslash nolimits}}_{k=1}^{n}\left|e_{i,j}x_{j,k}\right|$	A feature selection method using improved regularized LDA was proposed, which was verified with good performance on three DNA gene expression datasets.
[48]	2010	PCA	Fengxi Song et al.	$I_{j}=\sum {\mathrm{\backslash nolimits}}_{i=1}^{d}\left|e_{i,j}\right|$, (*d* = *s*)	PCA was used to rank original features and then key feature set is determined by FS, which was verified effective on three human face databases.
[51]	2016	PCA	I. Bhardwaj et al.	$I_{j}=\sum {\mathrm{\backslash nolimits}}_{i=1}^{d}\left|e_{i,j}\right|$, (*d* = *s*)	PCA was used to rank original features and then key feature set is determined by FS, which was verified effective on a fingerprint dynamics database.
[49]	2011	LDA	Fengxi Song et al.	$I_{j}=\sum {\mathrm{\backslash nolimits}}_{i=1}^{d}\left|e_{i,j}\right|$, (*d* = *s*)	LDA was used to rank original features and then key feature set is determined by FS, which was verified effective on three face databases.
[46]	2007	PCA/ICA	Z. Cataltepe et al.	$I_{j}=\sum {\mathrm{\backslash nolimits}}_{i=1}^{d}\left|e_{i,j}\right|$ (*d* ≤ *s*)	PCA/ICA was used to rank features respectively and then key feature set is determined by BS, which was verified effective on a corn fungi infection dataset.

Provided that a multivariate random vector $x=[f_{1},f_{2},...,f_{s}{]}^{T}$ is of *s* original features, and it is projected on a set of unit vector $\left\{e_{1},e_{2},...,e_{d}\right\}$ by linear transformation, so the transformed random vector $y=[y_{1},y_{2},...,y_{d}{]}^{T}$ can be represent as


$$y=[e_{1},e_{2},...,e_{d}{]}^{T}x$$ (1)

where


$$y_{i}=\sum {\mathrm{\backslash nolimits}}_{j=1}^{s}e_{i,j}f_{j},i=1,2,...,d.$$ (2)

and $e_{i,j}$ is the *j*-th component of the projection direction $e_{i}$. Intuitively, A bigger $\left|e_{i,j}\right|$ indicates more importance of the corresponding feature $f_{j}$ on the projection direction $e_{i}$, and the importance of $e_{i}$ can be denoted by contribution degree $C_{i}$,


$$\begin{array}{c} C_{i}=\frac{\lambda_{i}}{\sum_{j}\lambda_{j}}, \end{array}$$ (3)

where the $\lambda_{i}$ has different meanings in different linear-transformation technologies. For example, in PCA, $\lambda_{i}$ is the *i*-th largest eigenvalue of the covariance matrix of the observations of x. It denotes the sample variance on principle component $e_{i}$, and $\sqrt{\lambda_{i}}$ is the intensity of the component $e_{i}$; In LDA, $\lambda_{i}$ is the *i*-th largest eigenvalue of $S_{W}^{-1}S_{B}$ ($S_{W}^{-1}$ is the inverse of within-class scatter matrix and $S_{B}$ the between-class scatter matrix), and it denotes the Fisher separation or signal-to-noise ratio (SNR) on projection direction $e_{i}$. The detailed description and formulation about PCA and LDA can refer to the literature [47] and [50].

We revised the evaluation for feature relevance of $f_{j}$ as


$$I_{j}=\sum {\mathrm{\backslash nolimits}}_{i=1}^{d}C_{i}\frac{|e_{ij}{|}^{p}}{{\left({\left\|e_{i}\right\|}_{p}\right)}^{p}}$$ (4)

where p≠∞. The numerator $|e_{ij}{|}^{p}$ is the absolute value of $e_{i,j}$ to the power of *p*. The denominator $({\left\|e_{i}\right\|}_{p}{)}^{p}$ is the *p* vector norm of $e_{i}$ to the power of *p*, which is to normalize the numerator, and this makes the first issue come out, i.e., the normalization. The aim of the normalization is to guarantee the allocation of $C_{i}$ on $e_{i,j}$ is conserved, i.e., $C_{i}=\sum {\mathrm{\backslash nolimits}}_{j=1}^{s}C_{i}{\frac{|e_{ij}|}{\left\|e_{i}\right\|}}_{1}$. While, as shown in Table 3, a 2-norm normalization is equivalent to be adopted by considering that $e_{i}$ is usually normalized by 2-norm in the transformation process automatically, i.e., ${\left\|e_{i}\right\|}_{2}=1$, which causes an false allocation of $C_{i}$. For example, given a unit ei=[\raise0.7ex\({\sqrt 2 \)/22\nulldelimiterspace\lower0.7ex\(2\),\raise0.7ex\({\sqrt 2 \)/22\nulldelimiterspace\lower0.7ex\(2\)}}], then by the method of literature [47] the total allocated contribution $C_{i}$ would be $1.414C_{i}$, so obviously the increased 41.4% of $C_{i}$ is groundless. But in our formulation, the issue is solved by make the numerator ${\left|e_{ij}\right|}^{p}$ and its normalization term $({\left\|e_{i}\right\|}_{p}{)}^{p}$ matched.

Another issue is that the projection direction selection may affect the feature relevance evaluation. In literature [47] and [46], the authors noticed the effect on the final performance by selecting different projection directions to evaluate the feature importance, yet no practical method was presented to complete the selection. In the following subsection, we will present an implementation of the selection to calculate feature relevance by Eq. (4).

### 2.3. Interfered feature Elimination (IFE)

The IFE method used PCA and Pearson correlation coefficient to evaluate the feature relevance, and thereby eliminated the features badly interfered with the noise in unsupervised way. This method was built on a reasonable assumption that the ability of sensors to resist the actual noise is different since they are usually made with various materials and principles. Therefore, we could deduce that most sensors tended to point to directions that roughly consistent with the latent signal directions in feature space. Based on this deduction, we provided a specific implementation of the IFE method, which is as follows:

First, we normalized the original features by z-score standardization, respectively, so the variance of each original feature was scaled to 1. Then, we transformed the feature dataset by PCA, and the derived principal components with intensity bigger than 1 (which was equivalent to $\lambda_{i}\geq 1$) were considered to be consistent with the latent signal direction. Next, we used these components to calculate feature relevance by Eq. (4). The f 1, f 2, and f 3 denoted three features, respectively, and their intensity (module) was pre-scaled to 1. By PCA transformation of the three features, the principal components PC 1 and PC 2 with a bigger intensity were considered as be consistent with the latent signal direction. While the PC 3 with a smaller intensity was excluded because it might be badly interfered with.

Second, we sorted the features in descending order according to their individual relevance. We believe that the feature with a higher relevance was more likely to be the key feature than the feature with a lower relevance. Therefore, the feature was excluded if it was not correlated with each feature that was higher than it in relevance. The specific algorithm flow of the IFE method is listed in Table 4. The threshold $TH_{COR}$ was the minimum of the Pearson correlation, which is set below 0.4 empirically.

**Table 4:** Algorithm flow of the IFE method.

IFE method
**Input**: feature dataset X of *s* features and *n* observations. **Parameter:** A threshold of Pearson correlation coefficient TH_COR_. **Output**: ordered key-feature set S. **Procedure**: 1. Apply z-score standardization on each feature of X; 2. Exclude the principles with intensity bigger than 1; 3. Rank the *s* features in descending order of corresponding relevance calculated by Eq. (4), and an ordered feature set O is obtained; 4. Initial feature set S with $S_{1}=O_{1}$; 5. **for** i=2:s **for** j=1:end calculate Pearson correlation coefficient CoeffPearson(Oi,Sj)/CoeffPearson(Oi,Sj)σoi\nulldelimiterspaceσoiσsj; **end** **if** $all\left|\rho_{o_{i}}\left(j\right)\right|\leq TH_{COR}$, $O_{i}$ is excluded; **else** $S_{end+1}=O_{i}$; **end** **end**

### 2.4. Feature Group Selection (FGS)

**Fig 1:**
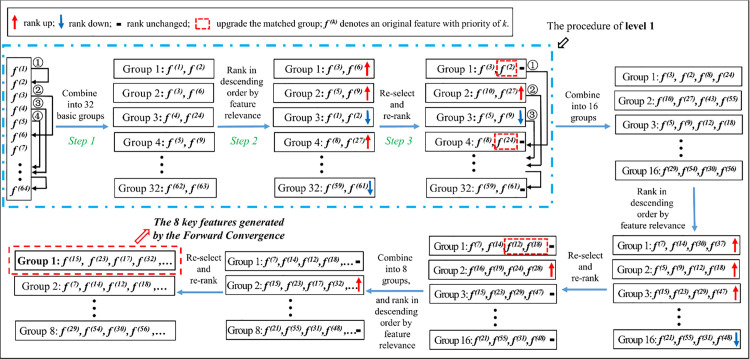
Schematic diagram of FGS.

An implementation of FGS method was drawn in Fig 1, which was to determine a combination of 8 features from 64 sensor features. Based on the specific implementation, the FGS method was elaborated as below.

**Fig 2:**
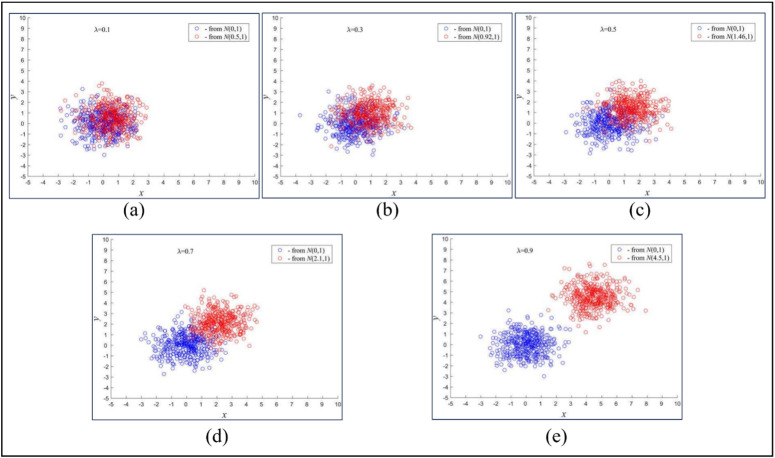
Random points from norm distribution with different lambda.

In the first step, we ranked the 64 features in descending order of their individual relevance calculated by regularized LDA (rLDA) [52]. In Step 1, feature *f* ^(1)^ was first assigned as a chooser group (a special group with one feature) to select its partner group from the remaining 63 groups (*f* ^(2)^ to *f* ^(64)^). The selected partner group should make the combination of the two groups achieving the maximum Fisher score calculated as Eq. (5),


$$\begin{array}{c} Score_{Fisher}\left(i\right)=\lambda_{i,1}+\sum_{k\geq 2,\lambda_{i,k}\geq TH_{F}}\lambda_{i,k}, \end{array}$$ (5)

where was the eigenvalue of the j-th projection direction of the combination of the chooser group and partner group i derived from rLDA. The threshold $TH_{F}$ was used to exclude the direction that was not effective for classification. As shown in Fig 2 (a)–(e), five scatter diagrams were draw with gradually increasing the distance between the centers of the two categories. In each of the diagrams, the 400 points were randomly chosen from a normal distribution with a standard deviation of 1. It was clear that the two categories become separable when the λ was bigger than 0.5. Therefore, it was appropriate to set $TH_{F}$ to about 0.5. Then, similarly, we assigned the subsequent single feature groups picked out the partner groups, respectively, and obtained 32 feature groups.

In the second step, an iterative ranking and selecting procedure were conducted in Steps 2 and 3. In Step 2, the 32 feature groups were ranked in descending order of their respective Fisher score. In Step 3, once found that the chooser group could not keep its previous rank when taking with the current partner group, a check procedure was conducted. For example, after Step 2, the previous first group {*f* ^(1)^, *f* ^(2)^} dropped to the third place and {*f* ^(3)^, *f* ^(6)^} moved up to the top, which was marked in red solid box in Fig 2,

In the third step, we first conduct the crossover selection to check the procedure. We extra calculated the score of {*f* ^(3)^, *f* ^(2)^} and {*f* ^(3)^, *f* ^(1)^}, respectively, and assigned *f* ^(2)^ or *f* ^(1)^ as a new partner group of *f* ^(3)^ if the new combination achieved a higher Fisher score. Then we conduct the mutation selection by extra calculating the score of {*f* ^(6)^, *f* ^(2)^} and {*f* ^(6)^, *f* ^(1)^}, respectively, and assigning *f* ^(2)^ or *f* ^(1)^ as a new partner group of *f* ^(6)^ if the new combination achieved a higher Fisher score. As a result, the group {*f* ^(3)^, *f* ^(2)^} achieved a higher score than {*f* ^(3)^, *f* ^(6)^}, so it was the new top group. Moreover, the feature *f* ^(6)^ was assigned to be the partner group of *f* ^(1)^.

When the check procedure to all the feature groups was finished, we ranked the new groups according to the new scores, and continued to conduct the procedure until the rank was not changed. In this setting, a feature group was given the right to select its partner according to its current rank but not to possess the partner. Similarly, in Stages 2 and 3, the group size was gradually enlarged to four and eight, and a group of 8 key features was formed on the top. Finally, a routine back sequential feature elimination (BSE) procedure was conducted, which iteratively eliminated a feature from the 8 key features and added a new feature until the Fisher score was not significantly increased.

### 2.5. Protocol and validation

Eight methods of the state of the art were used as benchmarks, i.e., FSASL (Feature Selection with Adaptive Structure Learning) [53], MCFS (Multi-Cluster Feature Selection) [54], UFSOL (Unsupervised Feature Selection with Ordinal Locality) [55], LASSO [56], Relief-F [57], SVM-RFE (Support Vector Machines with Recursive Feature Elimination) [58], mRMR (max-Relevance Min- Redundancy) [59], and RST (Rank Sum Test) [60,61]. For the validation on two bacteria datasets, the size of the optimized sensor combination was set to eight. Besides, we added six public gene expression profiling datasets for validation, including Srbct [62], ULC [63], Brain [64], Breast [65], Lymphoma [66], NCI [67], which was to increase the reliability of the validation.

Although our sensor array optimization method is Specifically proposed for the e-nose that applied to wound infection detection, the production and collection of our bacterial datasets are time-consuming and rare. Similar to the experiments on our bacteria datasets, we further did the experiments on the six public gene expression profiling datasets to verify the effectiveness of our proposed method. Gene expression profiling is the overall activity (the expression) of hundreds or thousands of genes, which is also known as gene fingerprint. A set of gene expression data can be processed by feature selection algorithm to find a good feature (expression) combination to better understand the information of these data. The size of the selected feature combination was also set to eight since the small feature combination can be perceived as the basis of any desired big feature combination. The description of the structure of the involved eight datasets is listed in Table 5.

**Table 5:** Structure description of the eight datasets.

Dataset	Class	Samples	Features_1_
Data1	8	428	90
Data2	8	320	90
Srbct	4	63	2308
ULC	9	675	147
Brain	5	42	5597
Breast	3	95	4869
Lymphoma	3	62.	4026
NCI 60	8	61	5244

For algorithm validation, to reduce the risk of overfitting, we used the protocol of 20 times 5-fold cross-validation instead of the common protocol of 10 times 10-fold cross-validation. We believed that the risk of overfitting could be greatly reduced by doubling the number of random tests and halving the number of partitioned subsets. Based on the adopted protocol, algorithm accuracy was calculated by averaging the 100 random recognition rates. Moreover, Least Squares Support Vector Machines (LSSVM) was relied as the classifier under the setting of RBF kernel and grid search for parameter optimization. The codes were executed at MATLAB 2024b on the Windows operating system with Intel(R) Core (TM) i7-4790K.

**Fig 3:**
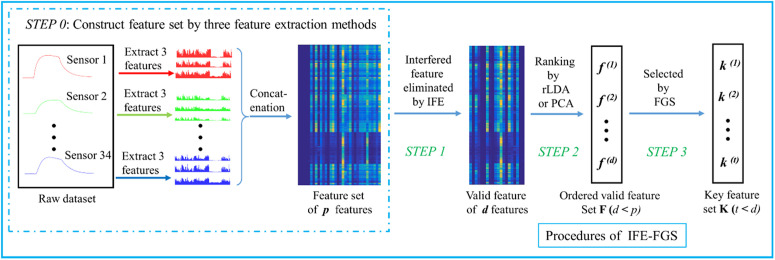
Overview diagram of the IFE-FGE on the bacteria datasets.

It needs to be explained that, for our application of bacteria detection, the optimization on the bacteria datasets consists of four steps, as shown in Fig 3. Initially, the respective performance of seven features common in the e-nose community [68] is investigated, and then three good features are selected to construct the feature dataset. In step 0, the three features are extracted on the 30 gas sensors, so totally 90 original features are obtained. In step 1, the IFE method is used to eliminate the interfered features. And in step 2, the remainders are ranked by the Fisher score of rLDA. Finally, the FGS method and BSE procedure are applied to determine the key feature set.

## 3. Results and discussion

### 3.1. Initial performance of the e-nose without SAO

A good initial performance is crucial to the result of the experiment. We did several experiments and then extracted seven popular e-nose features on the two bacteria datasets, including max response (Max), max amplitude (Amplitude), peak area, max slope, rising area, declining area, and purging area, and we gave a brief description of each of the features in Table 6. Based on 20 times 5-fold cross-validation, we investigated each feature’s performance separately on the two bacteria datasets. The investigation results (Table 6) showed that the Max feature performed better than other features, and achieved an accuracy of 88.64% on dataset 1 and 90.02% on dataset 2. Therefore, the two accuracies of the Max feature were considered as the initial performance of the e-nose on the two bacteria datasets before SAO.

**Table 6:** Initial performance of the e-nose with the use of all sensors and single feature extraction method.

Feature	Symbol	Description	ARR_dataset 1_	ARR_dataset 2_
Max	$V_{\max}$	The max response on sampling phase.	88.64/3.46	90.02/3.40
Amplitude	$V_{amp}$	$V_{\max}$ minus the average response on baseline phase.	80.16/3.84	81.02/4.84
Peak area	$V_{peak}$	The area enclosed by response curve and time axis on sampling phase.	80.05/4.39	78.88/4.63
Max slope	$V_{maxs}$	The max slope on sampling phase.	73.12/4.24	77.34/5.42
Rise Area	$V_{rise}$	The area enclosed by response curve and time axis from start of sampling to the time of max response slope.	51.93/4.98	51.00/4.87
Decline Area	$V_{decl}$	The area enclosed by response curve and time axis from time of max response slope to the time of max response.	71.04/4.10	71.12/5.01
Purging Area	$V_{purg}$	The area enclosed by response curve and time axis on purging phase.	72.15/3.68	72.10/4.77

### 3.2. Performance validations on the custom bacteria datasets and six public datasets

**Fig 4:**
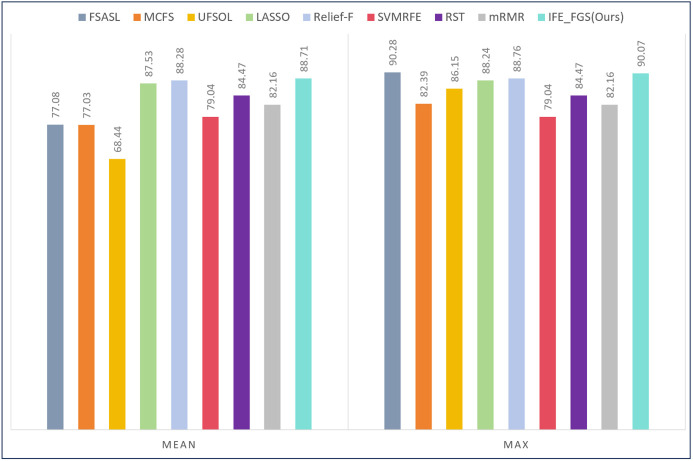
The experimental results of all the involved algorithms on the bacteria datasets 1.

**Fig 5:**
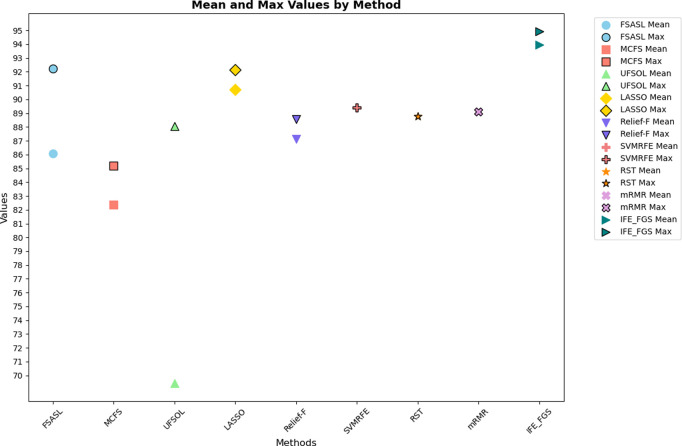
The experimental results of all the involved algorithms on the bacteria datasets 2.

**Fig 6:**
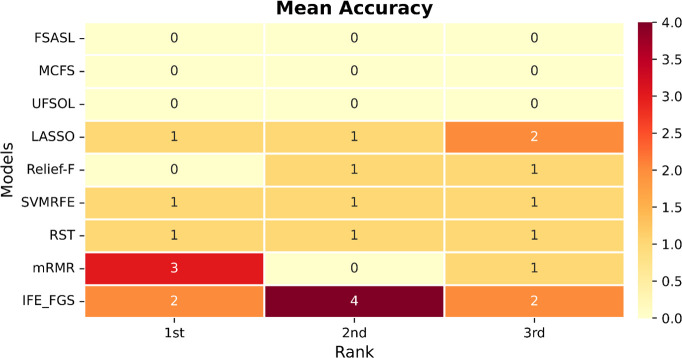
Heatmap matrix (Mean accuracy) for rank statistics of the validation results on the eight datasets.

**Fig 7:**
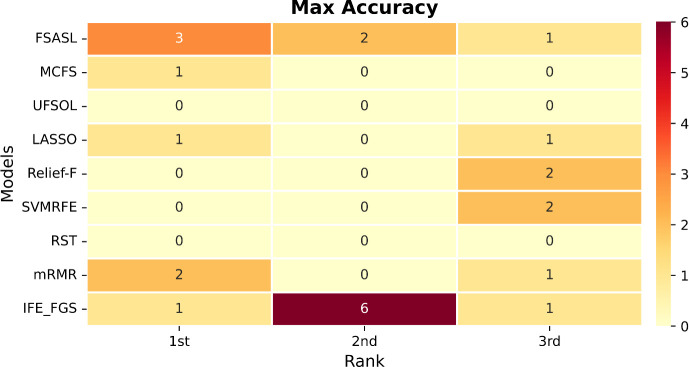
Heatmap matrix (Max accuracy) for rank statistics of the validation results on the eight datasets. *The cell denotes the number of corresponding rank (each column) achieved by the model (each row) on the results of the eight datasets.

The parameter configuration of the involved methods is listed in Table 7, and validation results of bacteria datasets 1 and bacteria datasets 2 are shown in Figs 4 and 5, respectively. The results of all the methods on the six public gene datasets are shown in Table 8. After statistics of the validation results listed in Figs 6 and 7, respectively. Moreover, two novel and interesting conclusions can be deduced through contrastive analysis as below.

**Table 7:** Experiment results of all the involved algorithms on the two bacteria datasets and six famous public datasets.

FSASL	MCFS	UFSOL	LASSO	Relief-F	SVMRFE	RST	mRMR	IFE-FGS
α: 0.005, 0.01, 0.05, 0.1..	P: 3, 4, 5	α:$10^{-4}$, $10^{-2}$, $10^{0}$, $10^{2}$, $10^{4}$.	λ: 1, 5, 10, 15, 20, 25.	k: 3, 4,5.	__	_	_	TH_F_: 0.50, 0.52, 0.54, 0.56, 0.58, 0.60.
β: 10^−4^, 10^−2^, 10^0^, 10^2^, 10^4^.	K: 1, 2, 4, 6, 8.	β: 10^−4^, 10^−2^, 10^0^, 10^2^, 10^4^.	_	_	_	_	_	$TH_{COR}$:0.10, 0.15, 0.20, 0.25, 0.30, 0.35,0.40.
γ: 10^−4^, 10^−2^, 10^0^, 10^2^, 10^4^.	_	_	_	_	_	_	_	_

**Table 8:** The experimental results (Mean) of all the involved algorithms on six public datasets.

	SRBCT	ULC	Brain	Breast3	Lymphoma	NCI 60
FSASL [53]	51.10/ 7.36	75.62/ 7.81	56.88/ 7.89	49.22/ 4.03	94.30/ 3.29	39.59/ 5.02
MCFS [54]	57.32/ 8.94	73.17/ 3.06	51.57/ 8.62	46.38/ 3.19	83.48/ 7.50	37.92/ 6.42
UFSOL [55]	66.69/ 5.05	75.97/ 2.40	30.59/ 5.26	43.67/ 2.26	73.25/ 7.67	17.81/-
LASSO [56]	67.09/ 1.79	80.37/ 0.42	60.01/ 1.02	**56.69**/**1.59**	94.75/ 0.18	35.05/ 2.11
Relief-F [57]	88.41/ 0.46	77.48/ 0.10	50.14/ 2.21	45.68/ 0.73	94.18/ 1.68	39.30/ 1.19
SVMRFE [58]	70.34/-	79.21/-	37.34/-	**56.00**/-	**96.77**/-	36.41/-
RST [60]	83.31/-	79.36/-	62.03/-	52.94/-	**96.68**/-	**48.03**/-
mRMR [59]	**95.38**	**82.13**/-	**63.31**/-	52.83/-	94.39/-	44.71/-
IFE_FGS (Ours)	**92.83**/ **0.66**	**80.61**/ **0.39**	**62.58**/ **1.69**	54.28/ 1.27	96.22/ 0.44	**45.87**/ **1.36**

*The cell of column “Mean” denotes the mean/standard deviation of the accuracies over model parameters of the corresponding model. When there is no parameter in a model, such as mRMR, or a model generates the same feature set over its parameters, the standard deviation is not exist, which is denoted by “-”;

From Fig 4, we can see that the IFE-FGS method achieved the best results both in mean accuracy and max accuracy on the bacteria datasets 1. To be specific, our proposed method obtained 88.71% accuracy, which was more than 20% higher than that of the UFSOL method. Although Relief-F got the second-best accuracy rate (88.28%), it still lagged behind us by 0.43%. For the experimental results in max accuracy, though the FSASL method could get 0.2% higher than ours, the mean accuracy of it is obviously poor compared with ours (77.08% vs 88.71%), which indicates that it is so susceptible to parameters. Besides, the accuracy rates of the rest of the seven compared methods did not exceed 89%. Therefore, it could be demonstrated that our proposed method is not only effective but also robust to parameters.

Different from the experimental results on bacteria dataset 1, the experimental results on bacteria dataset 2 illustrated that the advantages of our proposed method would be further enhanced. In Fig 5, we can see that the IFE-FGS method achieved the best performance (93.95%) in mean accuracy, which was 3.24% higher than the second-best method and as much as 24.71% ahead of the last one. A similar situation also occurred in the mean accuracy results. Our method obtained 94.94% accuracy, 2.7%, and 2.8% higher than the second-best and third-best methods. Comparing this with the initial performance of the current e-nose system indicates that the IFE- FGS method has effectively fulfilled the sensor array optimization target for the electronic nose applied to wound bacterial detection, which could significantly reduce the cost of sensors while improving system accuracy.

We further did the experiments on the six public gene expression profiling datasets to verify the effectiveness of our proposed method. From Tables 8 and 9, we can see that the IFE-FGS method took four second-places, two thrid-places in mean accuracy, and five second-places in max accuracy. Although we were specifically designed for the electronic nose dataset rather than the genetic dataset, our method has achieved acceptable results and demonstrated the possibility of its migration to other application scenarios.

**Table 9:** The experimental results (Max) of all the involved algorithms on six public datasets.

	SRBCT	ULC	Brain	Breast3	Lymphoma	NCI 60
FSASL [53]	65.89/ 14.06	**81.86**/ **2.91**	**69.39**/**13.30**	55.11/ 9.41	**97.96**/ **4.07**	48.86/11.39
MCFS [54]	74.19/ 11.11	77.12/ 3.24	62.94/14.13	52.00/ 8.76	93.82/ 6.07	**52.08**/**11.72**
UFSOL [55]	76.31/ 10.83	79.58/ 3.42	38.43/12.55	48.41/ 9.80	95.18/ 5.97	17.81/ 9.72
LASSO [56]	68.93/ 14.22	80.81/ 3.92	61.38/19.15	**58.56**/ **13.14**	94.92/ 5.14	37.72/12.10
Relief-F [57]	88.95/ 9.54	77.58/ 3.54	52.38/17.36	46.52/ 10.26	95.53/ 6.17	40.64/13.40
SVMRFE [58]	70.34/ 15.68	79.21/ 3.07	37.34/16.00	56.00/ 10.06	96.77/ 5.13	36.41/12.53
RST [60]	83.31/ 10.60	79.36/ 3.25	62.03/16.73	52.94/ 9.72	96.68/ 5.15	48.03/11.31
mRMR [59]	**95.38**/ **5.55**	**82.13**/ **3.00**	63.31/15.85	52.83/ 10.30	94.39/ 6.02	44.71/14.15
IFE_FGS (Ours)	**94.20**/ **6.22**	81.21/ 3.01	**65.87**/**14.54**	**56.88**/ **10.43**	**96.96**/ **5.30**	**49.35**/**11.20**

**The cell of column “Max” denotes the max of the accuracies over model parameters of the corresponding model, and the subscript denotes standard deviation of the 100 random test results when achieving the max accuracy.

In Figs 6 and 7, we can see that the IFE-FGS method showed consistency of effectiveness and achieved excellent performance on all the eight datasets, which took 2 first-places, 4 second-places, and 2 third-places in mean accuracy; 1 first-place, 6 second-places, and 1 third-place in max accuracy. This means that the IFE-FGS method has a distinct advantage in [53] model stability, which is a benefit of the group-based strategy of FGS because it can generate a stable structure of key feature sets by binding features in groups. However, none of the other methods can maintain effectiveness and usually fluctuate in accuracy when dealing with different datasets.

### 3.3. Comprehension of the feature recommendation by IFE-FGS

The above experimental results not only show the superiority of our proposed method, but also explore two interesting discoveries. 1) Each recommended feature set involved two or three types of features, the case that all eight features come from one type, such as $V_{\max}$ (which is the best one in our application) has never happened. It is the demonstration of sensor cooperation at the feature level. 2) Some sensors are always recommended, which are highlighted in Table 10. We may safely draw three new and valuable conclusions by summarizing the known volatiles of the three bacteria from previous research [69–72] listed in Table 11.

**Table 10:** Characteristics of the sensors recommended by IFE-FGS under limit of eight features.

No.	Model number	Type	Sensitivity	Target gases	Detection range
6	MS1100	MOS	High sensitivity to **toluene**, **formaldehyde**, **benzene, ethanol**.	VOCs, smoke, organic compounds	ppm level
8	MQ137	MOS	High sensitivity to **ethanol**, **NH**_**3**_, H_2_.	NH_3_, H_2_	NH_3_ 10-300 ppm
18	TGS822	MOS	High sensitivity to organic solvent vapors such as **ethanol**.	Organic solvent vapors	Ethanol 50 - 5000 ppm
20	GSBT-11	MOS	High sensitivity to indoor pollutants such as **formaldehyde, toluene, benzene, ethanol**.	VOCs, smoke, organic compounds	ppm level
26	MQ138	MOS	High sensitivity to **benzene, toluene**, methanol, **ethanol, formaldehyde, acetone.**	Organic solvent vapors	Benzene 1–100 ppm, toluene 10–100 ppm, methanol 5–100 ppm, ethanol 30–300 ppm, formaldehyde 1–10 ppm
30	TGS826	MOS	High sensitivity to **NH**_**3**_.	NH_3_	NH_3_ 30–300 ppm
34	TGS2602	MOS	High sensitivity to odorous gases such as **NH**_**3**_, H_2_S, **toluene, ethanol.**	Air pollutants such as ammonia toluene, H_2_S, ethanol, etc.	Ethanol 1 - 30 ppm

*The characteristics are referenced from official user manual of involved manufacturers.

**Table 11:** Known volatile organic compounds and gases emanated from the metabolite of the bacteria.

Bacteria	Volatile organic compounds and gases
*SA*	Ethyl alcohol, isobutanol, 1-undecene, methyl ketones, aminoacetophenone, **ammonia**, trimethylamine, 2, 5-dimethylpyrazine, isoamylamine, 2-methylamine, isopentyl acetate, acetic acid.
*PA*	Pyruvic acid, 2-nonanone, 2-hendecanone, 2-aminoacetophenone, dimethyldisulfide, dimethyltrisulfide, sulfocompound, methylbenzene, 1-hendecene, butanol, 2-butanone, isoamylol, isobutanol, isopentyl ester, methyl ketone, 2- heptanone.
*EC*	Ethyl alcohol, amyl alcohol, decyl alcohol, dodecyl alcohol, octanol, 1-propyl alcohol, benzpyrole, methyl ketone, lactic acid, acetic acid, succinic acid, formic acid, butanediol, aminoacetophenone, methanol, sulfuretted hydrogen, methyl mercaptan.

1. The algorithm always favors the sensors sensitive to the inorganic compound “ammonia,” which supports the previous research shown in Table 11 that ammonia is only found from the metabolite of Staphylococcus aureus (SA). Besides, the sensors with the ability to simultaneously be sensitive to ethanol, toluene, formaldehyde, or benzene are also preferred by the algorithm. Therefore, we have reason to believe that the chemicals belong to the biomarkers in the application.
2. The sensor only sensitive to oxycarbide, oxygen, sulfur dioxide, or hydrogen sulfide has never been selected by the algorithm, which indicates that they have no contribution in significance for the recognition.
3. From the perspective of engineer design for EN, we think that the selected sensor reveals the effective detection range of the targets. For ethanol, we found that the sensors TGS2602 with a detection range of 1–30 ppm, MQ138 of 30–300 ppm, TGS822 of 50–5000 ppm are strongly recommended by the algorithm, so they cover a detection range of 1–5000 ppm of ethanol, which should be considered as an effective range for the detection.

## 4. Conclusion

In this study, we presented the IFE-FGS method to optimize the sensor array for e-nose. Experimental results showed that the proposed methods could effectively reduce the number of sensors while improving the accuracy. Performance comparison with eight state-of-the-art methods on the two bacteria datasets and six public gene expression profiling datasets confirmed the effectiveness of the proposed methods. Besides, the SAO results helped us to further understand the different roles of the sensors in the application.

## Supporting information

S1 Data Accessible data. (RAR)
